# Enhancing Outcomes of Procedure Pairing With Next Generation Regenerating Skin Nectar With TriHex+ Technology

**DOI:** 10.1111/jocd.70556

**Published:** 2025-11-26

**Authors:** H. Ray Jalian, A. Jay Burns, Nazanin Saedi, Christopher Robb, Roy G. Geronemus, Kian Karimi, Faiza Shafiq, Tiffany Robison, Lora Colvan, Alan D. Widgerow

**Affiliations:** ^1^ Rebecca Fitzgerald MD Dermatology Los Angeles California USA; ^2^ Resurrect Skin MD Dallas Texas USA; ^3^ Dermatology Associates of Plymouth Meeting, P.C Plymouth Meeting Pennsylvania USA; ^4^ Skin and Allergy Center Spring Hill Tennessee USA; ^5^ Laser & Skin Surgery Center of New York New York New York USA; ^6^ Rejuva Medical Aesthetics Los Angeles California USA; ^7^ Director of Clinical Research Alastin Skincare, Inc. a Galderma Company Carlsbad California USA; ^8^ Clinical Research Manager Alastin Skincare, Inc. a Galderma Company Carlsbad California USA; ^9^ R&D Consultant Alastin Skincare, Inc. a Galderma Company Carlsbad California USA; ^10^ Division Chief Research, Professor Plastic Surgery, Center for Tissue Engineering University of California Irvine, Chief Scientific Officer Galderma. Irvine California USA

## Abstract

**Background:**

Regenerating Skin Nectar with TriHex+ Technology (Nectar 2.0; Alastin Skincare Inc., Carlsbad, CA) is a next‐generation anhydrous serum reformulated with Octapeptide‐45 to improve skin healing and hydration. Clinical trials and case studies assessed the safety, effectiveness, and patient satisfaction of Nectar 2.0 across various aesthetic procedures, including nonablative and ablative laser treatments, as well as microcoring technology.

**Methods:**

A multimodal clinical approach was used, including a randomized, double‐blind, split‐face study for nonablative laser procedures, an in‐use study assessing skin barrier function and hydration, and case studies for ablative laser and Ellacor microcoring treatments. Investigator and subject assessments, standardized photography, and bioinstrumentation measurements (TEWL and Corneometry) were utilized to evaluate outcomes.

**Results:**

Nectar 2.0 showed superior outcomes in reducing edema and enhancing tactile roughness and fine lines in nonablative laser procedures. In ablative and microcoring treatments, faster healing, less crusting, and decreased exudation were noted, along with higher patient satisfaction compared to the control. Bioinstrumentation confirmed statistically significant improvements in skin barrier recovery and hydration over 14 days. The product was well tolerated.

**Conclusion:**

Regenerating Skin Nectar with TriHex+ Technology Serum (Alastin Skincare Inc., Carlsbad, CA) has proven to be a safe and effective periprocedural skincare solution that enhances healing, improves skin quality, and increases patient satisfaction across various aesthetic procedures. Its benefits are especially significant in deeper treatments, supporting its widespread use in clinical practice.

## 1 Introduction

Regenerating Skin Nectar with TriHex Technology Serum (Alastin Skincare Inc., Carlsbad, CA) is an anhydrous gel containing TriHex peptides. It is a leading physician‐recommended skincare regimen for preparing skin before (preconditioning), during, and after aesthetic laser procedures. This TriHex serum is often used for preconditioning of the skin prior to procedures, based on the concept also known as skin bed preparation [1]. The TriHex peptides and botanical formulation have proven effective in aiding skin healing and rejuvenation, especially after various aesthetic procedures, as shown in previous publications [2, 3]. Products containing this patented peptide and botanical combination are associated with decreased inflammation, faster healing, and overall improved results and patient satisfaction postprocedure [2].

A recent reformulation of the TriHex serum was undertaken to include an added Octapeptide‐45, a proprietary peptide that promotes the skin's production of high molecular weight hyaluronic acid (HA) and provides anti‐inflammatory and immunosuppressive properties [4]. HA also plays an important role in wound healing processes.

Ex vivo models of TriHex 2.0 demonstrated increased HA and elastin stimulation, along with additional benefits such as wound healing capacity, stem cell recruitment, DEJ strengthening, and fibroblast antisenescence [3]. Here, we present a multimodal approach to assess safety and tolerability, as well as overall patient satisfaction and outcomes of Regenerating Skin Nectar with TriHex+ Technology Serum 2.0—Nectar 2.0 (Alastin Skincare Inc., Carlsbad, CA).

## 2 Materials & Methods

### 2.1 Photography

All participants provided a signed consent for the use of their images.

### 2.2 NonAblative Laser Procedures Clinical Study

Standardized facial imaging, including red and brown channel photography was performed at the screening Preconditioning visit 1b, baseline pre‐ and postlaser procedure, and at all follow‐up visits. Photography was performed using either a VISIA imaging system (Canfield Scientific Inc., Parsippany, NJ) or a Canon EOS 6D with a Westcott FJ400 strobe. Three different views were captured, including a front view and 45‐degree angles on both the left and right sides of the face.

### 2.3 Ellacor Micro‐Coring Technology and Healing Case Study

Photography was performed using either a Canfield IntelliStudio with Mirror Imaging (Canfield Scientific Inc., Parsippany, NJ) and a Canon EOS T6i or a Canon EOS 6D with a Westcott FJ400 strobe (Canon, USA Inc.).

### 2.4 Ablative Laser Procedures Case Study

Standard facial imaging was performed at all time points, including pre‐ and postprocedure at baseline, using either a Quantificare LifeViz camera (Quantificare Inc., Cummings, GA) or a VISIA imaging system (Canfield Scientific Inc., Parsippany, NJ).

### 2.5 Procedures

#### 2.5.1 Nonablative Laser Procedures Clinical Study

A randomized, double‐blind, split‐face, comparator study evaluating the effects of preconditioning with a TriHex Technology Serum Nectar 2.0 for enhanced wound healing versus a comparator (VaniCream lotion) was conducted from September 2023 through July 2024. The study was approved by Veritas IRB Inc. (Montreal, Quebec, Canada), and all subjects provided informed consent prior to any study procedures. Participants underwent one Moxi or Halo Fractional Laser (Sciton, Palo Alto, CA) facial procedure. A supporting skincare regimen was provided to all participants for use throughout the study, including a cleanser (Ultra Calm Cleansing Cream, Alastin Skincare Inc., Carlsbad, CA) and sunscreens (SilkSHIELD SPF 30, Alastin Skincare Inc., Carlsbad, CA, or HydraTint Pro Mineral Broad Spectrum Sunscreen SPF 36, Alastin Skincare Inc., Carlsbad, CA).

#### 2.5.2 Ellacor Micro‐Coring Technology and Healing Case Study

Four patients underwent one Ellacor Micro‐coring technology (Cytrellis Biosystems, Woburn, MA, USA) procedure of the face for the treatment of skin laxity and rhytids, following a preconditioning period (10–39 days) using Nectar 2.0 Serum and supporting regimen products (Ultra Calm Cleansing Cream, Ultra Light Moisturizer, Soothe + Protect Recovery Balm, and SilkSHIELD SPF 30, Alastin Skincare Inc., Carlsbad, CA). All products were used daily pre‐ and postprocedure in accordance with standard of care, and except for one clinical site where Soothe + Protect Recovery Balm was used postprocedure only after applying Nectar 2.0 Serum for 72 h postprocedure. The procedure was performed at a 5% density on the lower face (2.5–3.5 mm depth), cheeks (2.5‐4 mm depth), periorbital and chin (1.5‐2 mm depth) areas. Skin core collection ranged from 6510 to 6850 cores. Anesthesia was achieved with nerve blocks (infraorbital and mental; buccal approach) and 1% lidocaine with epinephrine (28‐35 cc). Patients were seen for follow‐up according to the investigator's standard of care through week 4, and photography was collected.

#### 2.5.3 Ablative Laser Procedures Case Study

8 patients were randomized equally to 2 treatment cohorts across 2 clinical offices and were seen for follow‐up postprocedure on days 1–4, 7, 10 and 14. Treatment cohorts included the following Test Product(s) and supporting regimen (Ultra Calm Cleansing Cream, Ultra Light Moisturizer, Soothe + Protect Recovery Balm (or petroleum ointment for control cohort), and SilkSHIELD SPF 30, Alastin Skincare Inc., Carlsbad, CA) in accordance with the case study physician's standard of care; Cohort 1–4 patients used Nectar 2.0 Serum full face and Cohort 2–4 patients used a control (VaniCream) full face. Patients underwent a 14‐day preconditioning period before their procedure, either 1 full‐field Erbium laser treatment of the face or 1 ProFractional laser treatment of the face and Contour TRL (full field) for perioral lines. Photographs were collected at every visit, and patient satisfaction questionnaires were completed at each follow‐up visit postlaser procedure.

### 2.6 Barrier Function & Hydration Bioinstrumentation Study

Barrier repair effects and hydrating properties of Nectar 2.0 Serum were evaluated in 20 subjects over 14 days. Transepidermal water loss (TEWL) measurements were performed using a DermaLab Evaporimeter (Cortex Technology, Hadsund, Denmark) to evaluate volar forearm skin barrier function of tape‐stripped skin of the 5 min post‐tape strip for a baseline reading preproduct use, 30 min after a single application of Nectar 2.0, and post 3, 7, and 14 days of consecutive twice daily product use (100 mg per application).

Corneometer CM 825 (Courage and Khazaka, Germany) hydration analysis was performed in parallel on both tape‐stripped skin of the volar forearm and intact nontape‐stripped facial skin at the same intervals across 14 days. Measurements were performed in triplicate immediately after 1 application, and post 3, 7, and 14 days of consecutive twice‐daily use of the test product (1 pump on face).

### 2.7 Assessments

#### 2.7.1 Investigator Assessments

##### 2.7.1.1 Nonablative Laser Procedures Clinical Study

Investigators assessed clinical grading at screening, baseline, and EOS using a Facial Aesthetic Scale to evaluate Global Photodamage, Tactile Roughness, Fine Lines, Hyperpigmentation, and Skin Dullness (Radiance) using a 10‐point Facial Aesthetic Scale (0 = None, 1–3 = Mild, 4–6 = Moderate, and 7–9 = Severe).

Investigators performed a healing assessment of the treatment areas (both sides of the face) pre‐ and postlaser procedure to assess healing parameters (Erythema, Edema, Crusting, Exudation), using a 5‐point scale where 0 = None, 1 = Very Mild, 2 = Mild, 3 = Moderate, and 4 = Severe, at baseline, and at all follow‐up visits. A Global Healing rating for each side of the face was also evaluated, where 4 = Excellent, 3 = Very Good, 2 = Good, 1 = Fair, and 0 = Poor.

#### 2.7.2 Ablative Laser Procedures Case Study

Blinded investigator clinical grading was performed using a 5‐point scale (0 = None, 1 = Very Mild, 2 = Mild, 3 = Moderate, 4 = Severe) to evaluate healing parameters (Erythema, Edema, Crusting, Exudation, Global Healing) postlaser treatment, and clinical photography was collected at all timepoints from baseline postprocedure.

#### 2.7.3 Subject Assessments and Questionnaires

##### 2.7.3.1 Nonablative Laser Procedures Clinical Study

A Subject Self‐Assessment of Healing was performed at Baseline pre‐ and postlaser procedure, and at all follow‐up visits to self‐assess healing parameters (Redness, Swelling, Crusting, Pain, Heat, Dryness) on both sides of the face separately, using a 5‐point scale where 0 = None, 1 = Very Mild, 2 = Mild, 3 = Moderate, 4 = Severe. Subjects completed the Subject Self‐Assessment Aesthetic Questionnaire at the Preconditioning Phase Screening visit (1b) prior to product use, at Baseline (Day 0) prior to the laser procedure, and at the EOS visit (Day 7 or Day 10) to self‐assess clinical signs (Dark Spots, Uneven Skin Tone and Texture) for both the right and left sides of the face, using a 10‐point scale, where 0 = Absent (best possible condition), 1–3 = Mild, 4–6 = Moderate, 7–9 = Severe (worst possible condition).

Subject tolerability was assessed at home on the day of the Preconditioning Phase screening visit (1b), where participants completed a Subject Tolerability Assessment to grade the degree of burning, itching, or stinging/tingling experienced postapplication of each product using a 4‐point scale (0 = None, 1 = Mild, 2 = Moderate, 3 = Severe).

Subjects reported their perception and satisfaction of both treatment sides by completing the Subject Test Product Experience Questionnaire for each side of the face separately at Baseline prior to their laser procedure, and at the EOS visit (Day 7 or Day 10) by selecting 1 of 5 responses (Strongly Agree, Agree, Neither Agree Nor Disagree, Disagree, Strongly Disagree), to each of the following statements: (1) This product spreads easily; a little goes a long way. (2) I feel that the product improves my skin hydration. (3) My skin looks recovered. (4) The product conditions my skin. (5) The product soothes my skin. (6) I feel the product does not irritate my skin. (7) The product helps to improve the overall appearance of my skin. (8) My skin looks firmer. (9) My skin looks younger, rejuvenated. (10) I feel the product helps to improve my skin texture. (11) Appearance of fine lines/wrinkles looks reduced. (12) Make‐up applies better over the product. (13) This is a game‐changing product.

#### 2.7.4 Ablative Laser Procedures Case Study

Patients reported their perception and satisfaction with the assigned Test Product at each follow‐up visit beginning at day 1 postprocedure through follow‐up day 14 by selecting 1 of 5 responses (Strongly Agree, Agree, Neither Agree Nor Disagree, Disagree, Strongly Disagree), to each of the following statements: (1) This product spreads easily, a little goes a long way. (2) I feel that the product improves my skin hydration. (3) My skin looks recovered. (4) The product conditions my skin. (5) The product soothes my skin. (6) I feel the product does not irritate my skin. (7) The product helps to improve the overall appearance of my skin. (8) My skin looks firmer. (9) My skin looks younger, rejuvenated. (10) I feel the product helps to improve my skin texture. (11) Fine lines/wrinkles look less noticeable. (12) Make‐up applies over the product better. (13) The product helps heal my skin after my laser procedure. (14) This is a game‐changing product. (15) I would recommend this product to others undergoing similar procedures. (16) Overall, I am satisfied with the product. Patients also reported Test Product usage in the 24 h prior to completing each questionnaire.

## 3 Results

### 3.1 Photography

#### 3.1.1 Nonablative Laser Procedures Clinical Study

**FIGURE 1:**
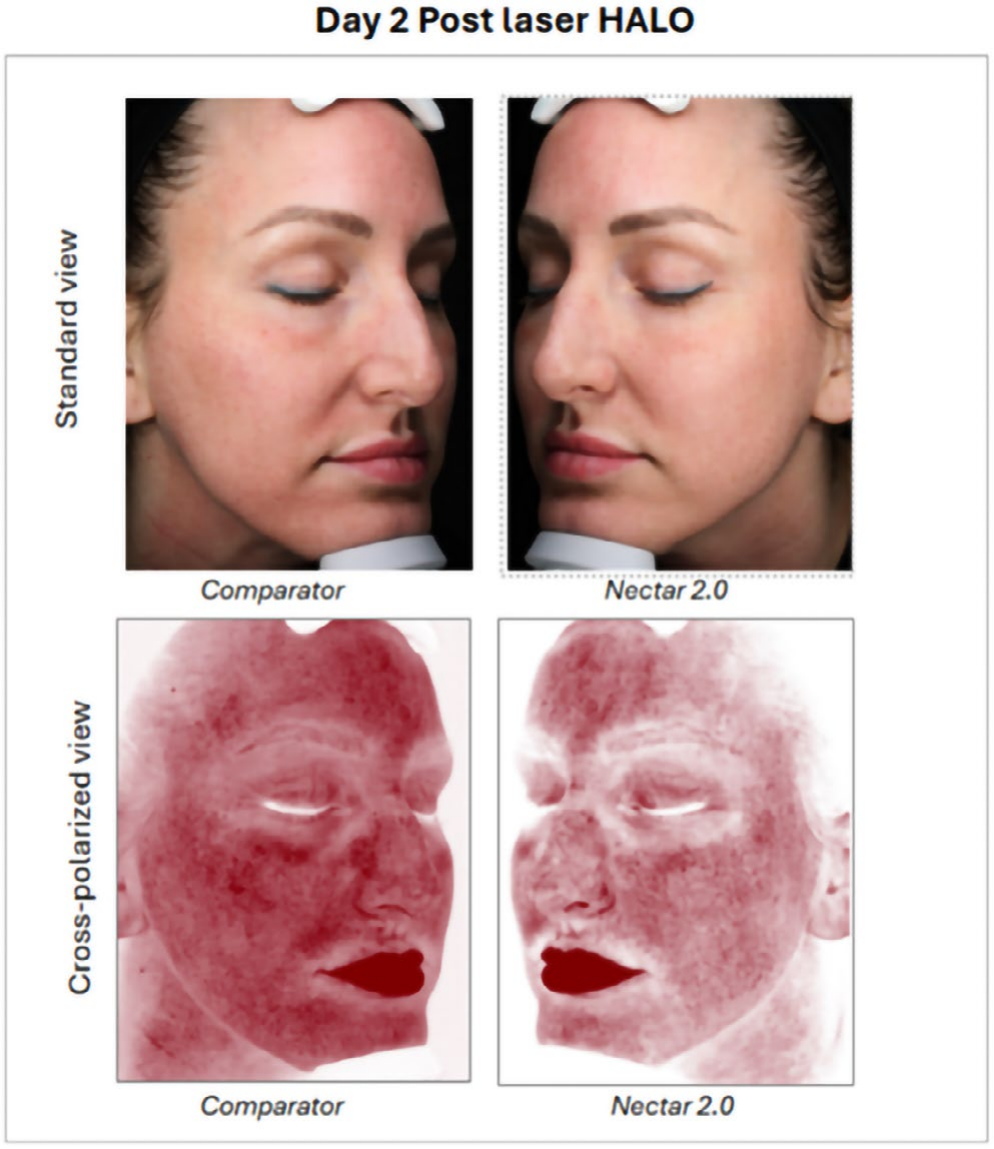
Female, age 38 years, Non‐Hispanic or Latino, Fitzpatrick Skin Type II. Standard imaging (Top) and red channel cross polarized (Bottom) at Day 2 post one Halo laser procedure of the face and split‐face use of the Comparator product (Right Side) and Nectar 2.0 Serum (Left Side), demonstrating markedly reduced erythema on the Nectar 2.0 treatment side vs. Comparator.

Standard imaging demonstrated results consistent with investigator clinical grading for the edema parameter, which was less pronounced on the Nectar 2.0 treatment side compared to the Comparator (VaniCream) side. Alternatively, the erythema and crusting parameters, despite the lack of statistically significant clinical grading results, both demonstrate lower severity on photography for the Nectar 2.0 treatment side vs. the Comparator (VaniCream) side (Figure 1).

#### 3.1.2 Ellacor Micro‐Coring Technology Healing Case Study

**FIGURE 2:**
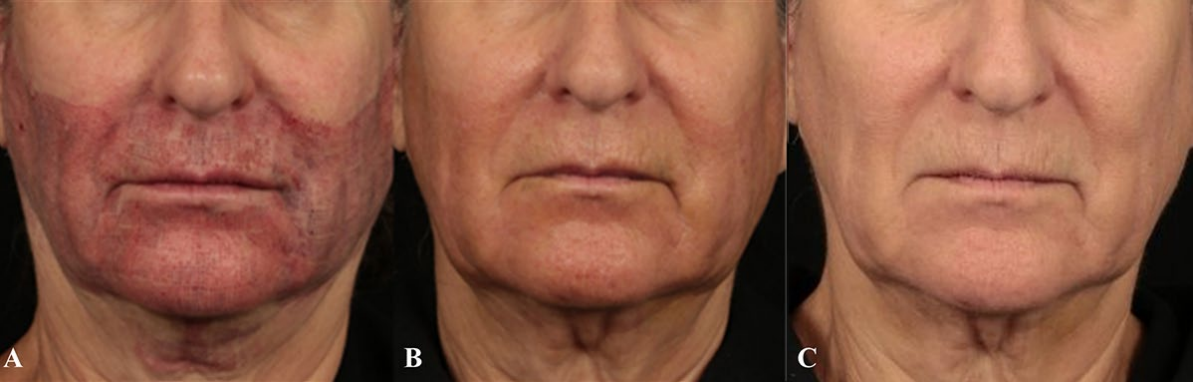
Female, age 66 years, Non‐Hispanic or Latino, Fitzpatrick Skin Type III. One Ellacor Micro coring technology procedure performed on face, submental and submandibular at 5% density and 8019 cores collected. Postprocedure photos Days 0 (A), 3 (B), 7 (C). Nectar 2.0 Serum and supporting regimen products were used 14 days preprocedure and through follow‐up.

Overall, 4 patients completed the case study. Mean age for completed subjects was 63.75 years (Range: 57–67 years), 100% (*n* = 4) were female. Study Subject Fitzpatrick skin type distribution included 75% (*n* = 3) were Type II, and 25% (*n* = 1) were Type III. Postprocedure healing captured through clinical photography demonstrated marked healing by Day 3 postprocedure, with resolution by Day 7 (Figure 2).

#### 3.1.3 Ablative Laser Procedures Case Study

**FIGURE 3:**
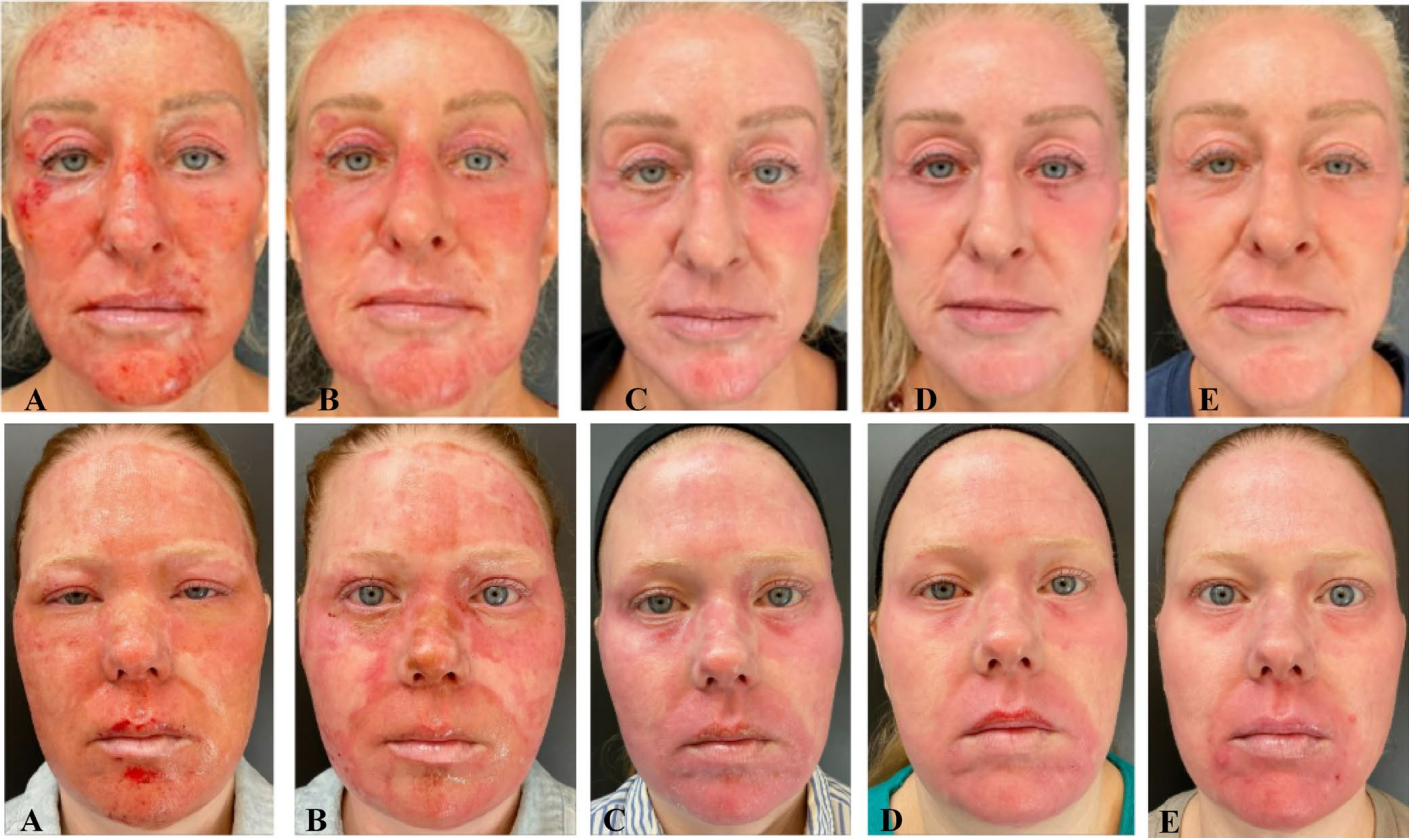
Females, Non‐Hispanic or Latino, ages 58 years, Fitzpatrick Skin Type III (Top), and 41 years, Fitzpatrick Skin Type II (Bottom), at Days 1 (A), 3 (B), 7 (C), 10 (D), 14 (E) post fully ablative laser procedure using either Nectar 2.0 Serum (Top) or Comparator (Bottom). Blinded Investigator global healing scores Day 1 postprocedure of 4‐Poor Healing were assigned for both patients, whereas a score of 0‐Excellent Healing (Nectar 2.0), and 1‐Very Good Healing (Comparator) were achieved at Day 14.

Standard photography revealed consistent postprocedure healing through the follow‐up period with the blinded clinical grading. Overall, improved healing and recovery for the Nectar 2.0 treatment patients compared to VaniCream (Comparator) were demonstrated, where notable healing improvements in edema, crusting, and exudation were achieved (Figure 3).

### 3.2 Investigator Assessments

#### 3.2.1 Nonablative Laser Procedures Clinical Study

Overall, 20 subjects completed the study, while 1 subject withdrew consent during the preconditioning phase. The mean age of completed subjects was 45.1 years (range: 33–70 years). Of these, 95% (*n* = 19) were female, and 5% (*n* = 1) were male. The Fitzpatrick skin type distribution included 5% (*n* = 1) Type I, 70% (*n* = 14) Type II, 15% (*n* = 3) Type III, and 10% (*n* = 2) Type IV. Participants underwent either one Halo (*n* = 15) or Moxi (*n* = 5) Fractional Laser (Sciton, Palo Alto, CA) procedure on the face. Halo laser treatments were performed at wavelengths of 1470 nm (depth 300–400 μm, 10%–35% density) and 2940 nm (depth 20–100 μm, 10%–26% density). Moxi laser treatments used 15–20 mJ and 9–15 passes.

Investigator clinical grading of facial aesthetic parameters showed a statistically significant greater improvement in fine lines on the Nectar 2.0 side (65%) compared to VaniCream (45%) at EOS from screening (*p* = 0.0455). Nectar 2.0 also achieved a greater improvement (77.8%) in tactile roughness at EOS from screening compared to VaniCream (68.4%), although this difference was not statistically significant. No other significant differences between the treatment sides were observed for global photodamage and skin dullness parameters.

Investigator healing assessments indicated that edema ratings were better after laser treatment, reaching statistical significance (*p* = 0.0421) by the follow‐up visit on Day 5. Facial edema fully resolved on both sides by Day 10 postlaser. The overall healing parameter favored Nectar 2.0 over VaniCream at all postlaser timepoints, but these differences were not statistically significant. No significant differences were observed in erythema, crusting, or exudation on either side, suggesting comparable results.

#### 3.2.2 Ablative Laser Procedures Case Study

**FIGURE 4:**
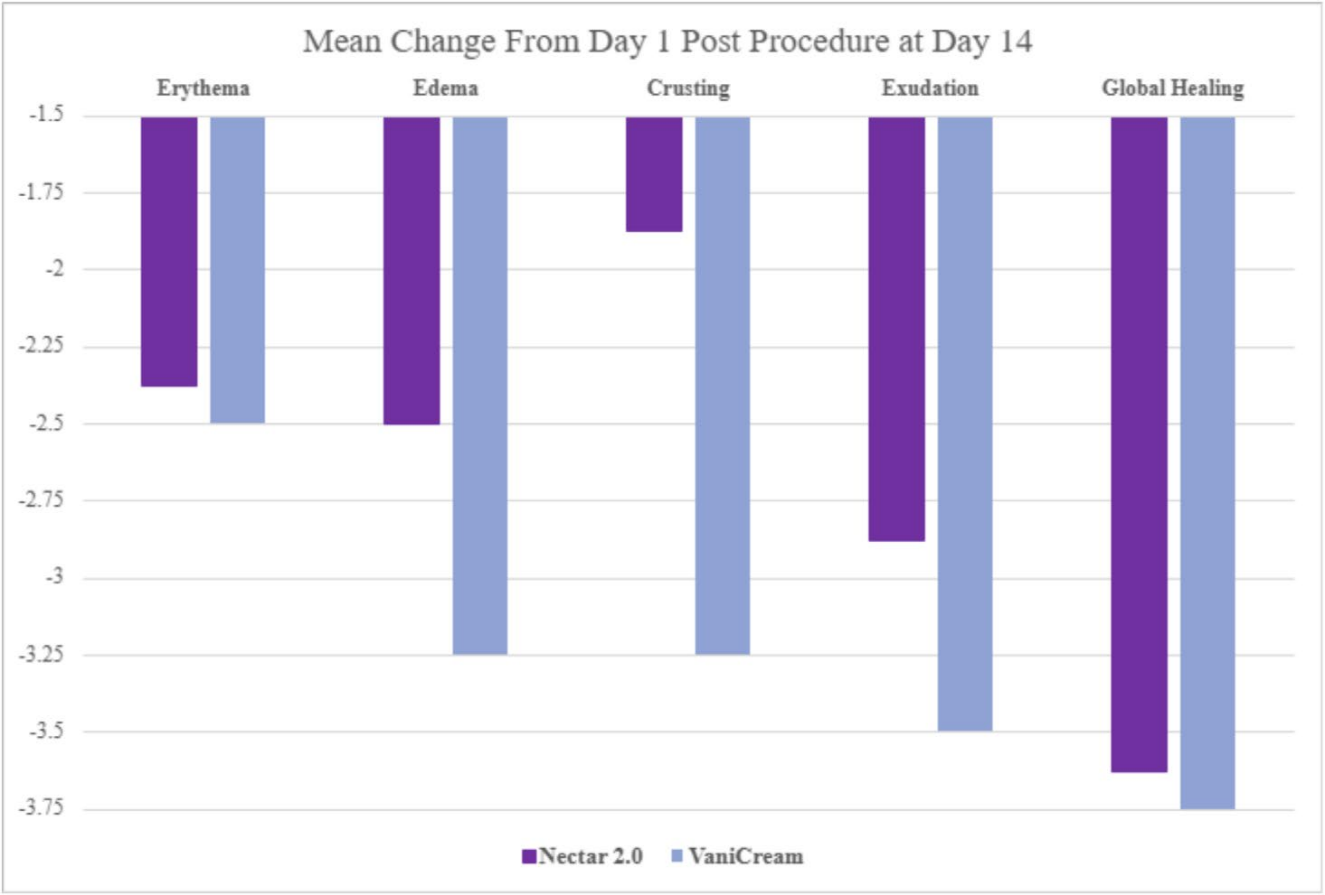
Blinded clinical grading mean change ratings for healing parameters at Day 14 from Day 1 postprocedure.

Blinded clinical grading of healing parameters revealed overall improved healing and recovery postprocedure for the Nectar 2.0 cohort compared to VaniCream, achieving superior mean improvements at Day 14 postprocedure for the edema (26.087%), crusting (53.6585%), and exudation (19.6078%) parameters. Modest superiority of Nectar 2.0 in mean healing change was observed for the Erythema (5.12821%) and Global Healing (3.38983%) parameters (Figure 4).

### 3.3 Subject Assessments and Questionnaires

#### 3.3.1 Nonablative Laser Procedures Clinical Study

Postprocedure use of either Nectar 2.0 or VaniCream compared to baseline demonstrated slightly shorter healing times (ranging from −0.05 to −0.40 days) on the Nectar 2.0 treatment side for all parameters (Redness and Swelling: −0.15 days, Crusting: −0.05 days, Pain: −0.35 days, Heat: −0.25 days, Dryness: −0.40 days), although the differences were not statistically significant. The healing differences between both treatment sides were determined overall to be similar for all parameters and no statistically significant differences were achieved.

The Subject Self‐Assessment Aesthetic Questionnaire revealed improvements from baseline preprocedure at EOS, achieving equivalence on both treatment sides for dark spots (66.7%) and tone (78.9%) parameters. The texture parameter, however, demonstrated greater improvements on the Nectar 2.0 (75%) treatment side compared to the VaniCream side (65%), although not statistically significant.

**FIGURE 5:**
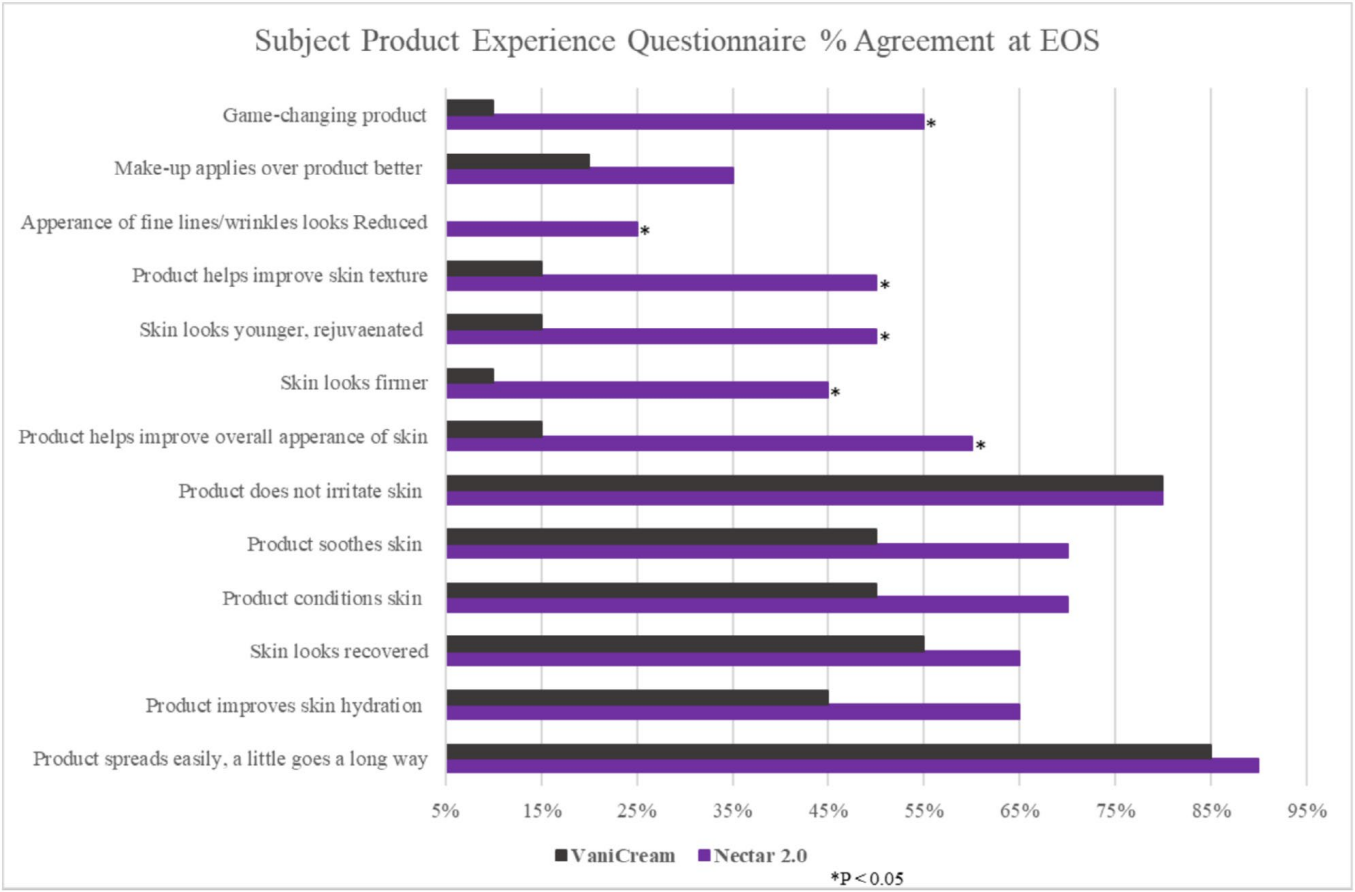
Subject Test Product Experience Questionnaire at EOS‐Percentage agreement.

Overall, the percentage of agreement was higher for Nectar 2.0 than VaniCream across all questions (at Baseline and EOS), except for “My skin looks younger, rejuvenated” at baseline, where both treatments were equal. The percentage of agreement was significantly higher for Nectar 2.0 at the EOS visit compared to VaniCream for the following questions: The product helps improve the overall appearance of my skin (60%; *p* = 0.0027). My skin looks firmer (45%; *p* = 0.0082). My skin looks younger, rejuvenated (50%; *p* = 0.0082). I feel the product helps improve my skin texture (50%; *p* = 0.0082). The appearance of fine lines/wrinkles looks reduced (25%; *p* = 0.0455). This is a game‐changing product (55%; *p* = 0.0027) (Figure 5).

#### 3.3.2 Ablative Laser Procedures Case Study

Patient questionnaires completed during follow‐up showed a general preference for Nectar 2.0 Serum over the Comparator for the following statements: This product spreads easily, a little goes a long way, my skin looks recovered, the product soothes my skin, I feel the product does not irritate my skin, the product helps improve the overall appearance of my skin, my skin looks younger and rejuvenated, I feel the product helps improve my skin texture, the product helps heal my skin after my laser procedure, this is a game‐changing product, and I would recommend this product to others undergoing similar procedures. Patients reported similar levels of favorability for both products regarding the following statements: the product conditions my skin, I feel that the product improves my skin hydration, my skin looks firmer, and make‐up applies better over the product.

**FIGURE 6:**
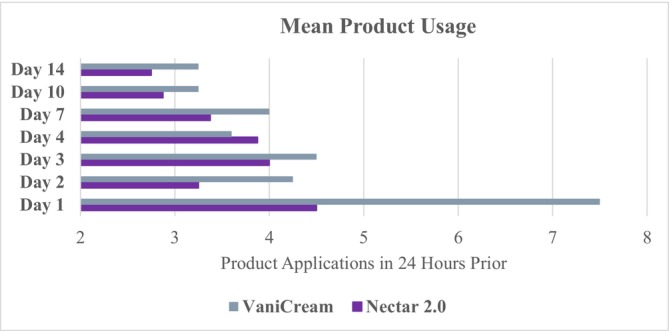
Patient reported product usage (applications) in the 24 h prior to each time point.

Patient reported less use of the assigned product in the 24 h before completing the questionnaire, with Nectar 2.0 Serum use being lower at each time point except for Day 4, when VaniCream use was lower. The same pattern was observed for the period prior to questionnaire completion, with Nectar 2.0 Serum use being lower at each time point except for Day 4, when VaniCream use was lower (Figure 6).

### 3.4 Barrier Function & Hydration Bioinstrumentation Study

**FIGURE 7:**
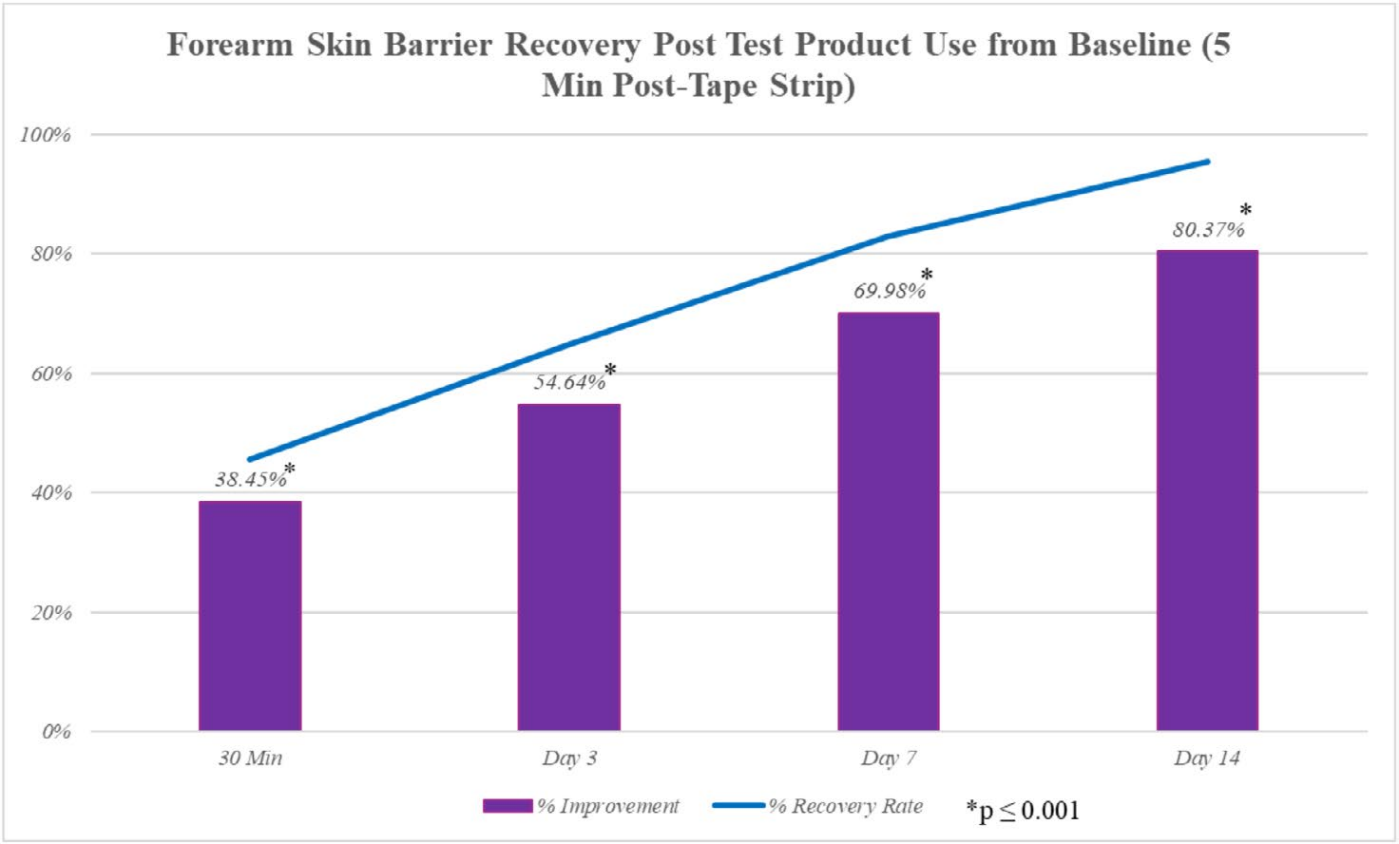
Forearm skin barrier TEWL analysis demonstrated improvements in barrier function and recovery at all timepoints postapplication compared to baseline.

Barrier function and hydration were evaluated in 20 subjects aged 21–60 years (mean age of 41 years), with 80% female (*n* = 16) and 20% male (*n* = 4). The group included 15% who reported self‐perceived sensitive skin (*n* = 3), and the Fitzpatrick skin type distribution was 10% type I (*n* = 2), 20% type II (*n* = 4), 30% type III (*n* = 6), and 40% type IV (*n* = 8). Forearm skin barrier function, assessed through TEWL analysis, showed statistically significant improvements in 100% (*p* ≤ 0.001) of subjects at all time points compared to baseline—measured 5 min post‐tape strip prior to product application. TEWL decreased by −38.45% at 30 min postapplication, −54.64% at day 3, −69.98% at day 7, and −80.37% at day 14. Skin barrier recovery followed similar trends, with recovery percentages improving at each time point, reaching 95.34% by day 14 and 100% of subjects showing improvement from baseline (Figure 7).

Statistically significant improvements in forearm skin hydration were observed starting 5 min after tape strip application, with a 13.95% increase at 30 min postapplication (*p* = 0.002). These improvements persisted through the end of the study at day 14, with hydration increases of 23.52% on day 3, 27.15% on day 7, and 28.59% on day 14 (all *p* ≤ 0.001), indicating skin repair and a return to a noncompromised state.

Corneometer hydration evaluation on noncompromised facial skin revealed significant improvements from baseline at post‐treatment day 3 (9.20%) (*p* = 0.039), day 7 (13.26%) (*p* = 0.041), and day 14 (7.23%) (NS) after continued product use.

## 4 Tolerability and Safety

### 4.1 Tolerability Assessments

Tolerability was assessed during the nonablative laser procedures clinical study on the day of the Preconditioning screening visit 1b, after the first at‐home application of both test products. It showed burning and itching in 5% (*n* = 1) of participants on both treatment sides, and stinging/tingling in 5% (*n* = 1) on the Nectar 2.0 side and 10% (*n* = 2) on the VaniCream side. Overall, no significant differences in tolerability were observed between the two treatment sides. No significant differences were found in tolerability between the treatment groups. Occasional, transient, and self‐limiting burning and moderate itching were associated with both products; however, no substantial differences in tolerability were identified between the two groups.

### 4.2 Adverse Events

Two adverse events (AEs) of rash (bilateral face, bilateral cheeks) were reported for the split‐face nonablative laser procedures at one clinical site for 1 study participant. Both AEs were reported for the product and comparator, and both resolved without intervention. No study test product related adverse events were reported during skin barrier function and hydration testing.

Overall, Nectar 2.0 Serum has demonstrated safety and tolerability for use immediately postprocedure and during the follow‐up healing period.

## 5 Discussion

For the past 10 years a new approach to periprocedure management has been introduced and successfully validated using TriHex Technology. The approach was based on wound healing principles, considering skin as a chronic wound constantly undergoing extrinsic and intrinsic stressors, placing it in a constant healing state. As such, one of the most important lessons learned from treating chronic wounds was that the wound needed preparation, wound bed preparation, prior to applying any topical therapy [5]. Adhering to that concept, “skin bed preparation” was introduced as a method of preconditioning the skin prior to a procedure. The purpose was to remodel the extracellular matrix (ECM), removing fragmented collagen, elastin, glycation end products, and senescent cells and replacing these degenerated proteins with newly formed replacements. The combination of peptides used with this technology proved adept at doing just that—recycling the matrix to produce a newly regenerated matrix that was conducive to cellular protein cross‐talk and efficient healing. This manifested as improved healing and optimized outcomes [2, 3, 4, 6, 7]. The TriHex Technology was recently updated to include Octapeptide‐45, which demonstrated new regenerative properties relating to the DEJ (Dermo Epidermal Junction), decreased senescent SASP (Senescence‐Associated Secretory Phenotype) effects, improved hydration and enhanced elastin regeneration [3]. This paper investigates the use of this advanced technology with multiple procedures. It examines the application of advanced technology across. These were selected to indicate the universal applicability of the formulation for periprocedure use across a broad spectrum of devices. These included split‐face superficial procedures (Moxi, Halo) to cohort comparisons of slightly deeper ablations using CO_2_ to full‐thickness coring of tissue (Ellacor), constituting the deepest device intervention. Through the years, it has been apparent that the deeper the process, the better this formulation performs. This is not surprising as re‐epithelialization or healing in superficial procedures takes place anywhere from a few hours to 1 or 2 days, making significant comparisons very difficult. Where healing takes place over 5 days to 2 weeks, TriHex+ Technology has maximal benefits. This is evident in the current study, too, where the most superficial nonablative procedures showed improvements in symptoms such as edema, but healing was not enough of an issue to show a real difference, although the TriHex+ group tended toward healing superiority and shorter times to healing when assessed by investigators. In these cases, improvements in symptoms and signs associated with the procedure were evident, and where the focus was placed, subjects reported their skin feeling younger, firmer, and more rejuvenated on the TriHex+ side. This is in direct contrast to the full‐thickness coring device that showed distinctive healing at 3 days postprocedure, very early in the recovery process, with healing ensuring protection against significant “spot” marks from delayed healing. This is an extremely important advantage for this type of device and procedure. This also validates the safety of this anhydrous product across a spectrum of devices.

In addition to procedure/device assessments, additional analyses were undertaken to gauge the difference in hydration and barrier function with the addition of Octapeptide‐45 to the TriHex Technology. Results of the TEWL analysis showed significant improvement in barrier function. The addition of Octapeptide‐45 has shown hydration benefits in other studies, and the combination showed added benefits on DEJ measurements. This was particularly evident when one observes the usage of the Nectar 2.0 vs. the competitor, which was far less, implying that patients were better hydrated, feeling less dryness with the Nectar 2.0.

Finally, in an additional study, histological assessments were carried out on patients preconditioned for surgery, comparing TriHex+ Technology on one side to vehicle alone on the opposite side (in a separate submission JoCD). Dramatic results were seen in some of these cases with obvious reversal of solar elastosis, remodeling of the ECM and reversal of effacement of the DEJ with new rete pegs evident. To supplement these obvious changes seen in H&E views, Herovici stains showed significant neocollagenesis with early new fibers present, while Movat stains and CD44 demonstrated neoelastogenesis and marked stimulation of HA production. This was in direct contrast to the vehicle side, which showed minimal changes in all stains assessed.

Cumulatively, these documented changes suggest that this new formulation has applicability to all types of devices, the more superficial procedures benefiting from reduced symptomatology and improved subject responses to the procedures, while deeper processes showed distinct healing advantages.

## 6 Conclusion

Regenerating Skin Nectar with TriHex+ Technology Serum (Alastin Skincare Inc., Carlsbad, CA) represents an advance in the TriHex Technology. The addition of an extra peptide has shown marked synergy both in preclinical tests and in clinical outcomes. As with previous experience, this technology really shines as the intervention becomes deeper and healing time is increased with procedures such as full‐thickness coring, showing dramatic healing advantages. Being nonaqueous in nature and formulated with a minimal number of preservatives, the tolerability is excellent and adverse events are minimal as opposed to some of the biologic products on the market. This formulation has been demonstrated to be safe and effective as a periprocedural skincare solution that enhances healing, improves skin quality, and increases patient satisfaction across a spectrum of aesthetic procedures. This marks an exciting new next generation of TriHex Technology in the periprocedure space.

## Author Contributions

A. Jay Burns – study investigator, paper writing contribution. Alan D. Widgerow – developed the science, analysis, paper writing. Christopher Robb – study investigator, paper writing contribution. Faiza Shafiq – study concept and design, paper writing and data analysis. H. Ray Jalian – study investigator, paper writing contribution. Kian Karimi – case study investigator, paper contribution. Lora Colvan – study design. Nazanin Saedi – study investigator, paper writing contribution. Roy G. Geronemus – case study investigator, paper writing contribution. Tiffany Robison – study design and management, data analysis, paper writing.

## Conflicts of Interest

Alan D. Widgerow (Chief Scientific Officer), Faiza Shafiq (Director Clinical Research), Tiffany Robison (Manager, Clinical Research) are all employees of Galderma. Lora Colvan is a part time consultant for Alastin Skincare Inc., a Galderma company.

## Funding

This study was supported by Alastin, a Galderma company.

## Data Availability

The data that support the findings of this study are available on request from the corresponding author. The data are not publicly available due to privacy or ethical restrictions.
